# ‘Ageing with an alcohol problem is not what I envision’: reclaiming agency in shaping personal ageing trajectory and recovery from alcohol problems

**DOI:** 10.1186/s12877-023-04573-y

**Published:** 2023-12-16

**Authors:** Wossenseged Birhane Jemberie, Fredrik Snellman, Malin Eriksson, Anders Hammarberg

**Affiliations:** 1 Department of Social Work, Umeå University, 901 87 Umeå, Sweden; 2 Centre for Demography and Aging Research (CEDAR), Umeå University, Umeå, Sweden; 3 The Swedish National Graduate School for Competitive Science On Aging and Health (SWEAH), Faculty of Medicine, Lund University, Lund, Sweden; 4 Centre for Psychiatry Research, Department of Clinical Neuroscience, Karolinska Institutet and Stockholm Health Care Services, Region Stockholm, Stockholm, Sweden

**Keywords:** Alcohol use disorder, Healthy aging, Aged, Quality of life, Recovery, Stigma, Alcohol treatment, Person-centered care, Alcohol

## Abstract

**Background:**

Eliciting and understanding older persons’ descriptions of their resources for healthy ageing and the interaction of these resources with alcohol use and alcohol problems can facilitate health promotion. It can also inform clinicians when identifying areas of recovery capital that present risks and strength for older people seeking alcohol treatment. The objective of this study was to illuminate the experiences and perspectives of older persons on ageing, alcohol use, treatment, and recovery from alcohol problems, as well as their understanding of healthy ageing.

**Methods:**

Eight men and two women, aged 61 to 73 years, with moderate drinking as a treatment goal and treated at an outpatient alcohol clinic in Sweden, participated in semi-structured audio-recorded virtual interviews. A qualitative content analysis examined the transcribed interviews.

**Results:**

Three themes were identified: “Tipping the balance”, “Staying behind a veil” and “Lifting the vail”. First, participants understood healthy ageing as a personal and multidimensional process that involved actively expanding, maintaining or adjusting to the resources needed to lead an active and meaningful life while preserving autonomy, dignity and independence for as long as possible. Second, most participants viewed moderate alcohol use as a contributor to healthy ageing. They sought treatment when their drinking became unsustainable and an immediate threat to their healthy ageing resources. Stigma, ambivalence and a lack of treatment options, however, contributed to delayed treatment. Third, the participants responded to treatment approaches that elicited their concern, incorporated their expertise and treatment and life goals, appreciated their autonomy and agency, and considered them partners in goal setting and decision making. Reduced drinking helped participants regain their agency and improved their healthy ageing capital which in turn catalyzed continuing recovery.

**Conclusions:**

Older persons in non-abstinent recovery perceive healthy ageing and alcohol recovery as personal and interacting multidimensional processes involving their agency to improve biopsychosocial functioning. Treatment approaches that recognize older persons’ desire for healthy ageing, incorporate their treatment goals and respect their autonomy are likely to be acceptable and effective.

**Supplementary Information:**

The online version contains supplementary material available at 10.1186/s12877-023-04573-y.

## Introduction

The World Health Organization (WHO) defines healthy ageing as *‘the process of developing and maintaining the functional ability that enables wellbeing in older age’* highlighting the ongoing interaction between individuals and their environments [1]. One modifiable environmental factor that detrimentally affects ageing trajectories is problematic alcohol use. Despite the increase in the number of older people with alcohol problems [2–4] and the resulting alcohol-related medical and psychosocial problems [5–7], the topic of healthy ageing in this demographic, however, remains relatively unexplored. Eliciting the resources that older people perceive as central to healthy ageing is crucial when investigating the availability of resources that may interact with alcohol use, alcohol problems and recovery. By addressing the interplay between alcohol use and ageing, and understanding how older persons negotiate their lived experiences, it may be possible to promote healthy ageing and reduce harmful alcohol use among older people.

A recent study by Kermel‐Schiffman and Gavriel‐Fried focused on the life experiences of older persons in abstinent recovery [8]. However, research suggests that more than half of older people with alcohol problems prefer moderate drinking as a treatment goal which underscores the need for exploring ageing, alcohol use, and recovery experiences among older people in non-abstinent recovery [9].

### Background

Researchers use various variables related to physiological, well-being, engagement, personal, and extrinsic factors to conceptualize healthy ageing [10–16]. These constructs are either researcher defined or reflect the perspectives of older persons [11, 17–19]. There is, however, a disagreement between the objective criteria and older people’s subjective understanding of healthy ageing. Older individuals often view healthy ageing as a process rather than an outcome and place more importance on psychosocial functioning than physical health [11, 20–22].

A positive self-perception of ageing is associated with preventive health behaviors and better health outcomes among older people [23]. One specific behavior that may be influenced by self-perception of ageing is alcohol use. According to Villiers-Tuthill and colleagues, older persons who have a positive outlook on ageing and feel that they have agency over their ageing experience are less likely to have harmful alcohol use and more likely to use alcohol in moderation [24]. In contrast, those who feel negatively about ageing are more likely to have harmful alcohol use [24].

Treatment coverage for alcohol problems is estimated only at 10–20% globally [25]. About 40% of adults who feel the need for treatment for an alcohol problem do not seek care because they are not ready to stop drinking [26]. In fact, abstinence may not be an attractive treatment goal for many individuals [27–30]. This suggests that abstinence, the primary measure of success in alcohol use disorder treatment, can be a deterrent to treatment seeking, especially for those who want to reduce their drinking without a commitment to abstinence [27, 29, 30]. Moreover, there may be discrepancies between the experiences of people in recovery and prescribed recovery goals that include abstinence. Many people in recovery may find these prescribed goals to be unrelatable to their personal experiences and insensitive to the individual differences and preferences that exist among people in recovery [31].

Additionally, the variables associated with recovery capital are heterogeneous among different population groups [32, 33]. This may be particularly true for older people, who may face a range of changes in their physical health, social networks, and cultural and social recovery capital due to alcohol use and ageing [34]. Because alcohol use, treatment goals, and recovery capital may differ among older and younger adults, it is important to conduct qualitative research with older people to understand their lived experience with alcohol use, treatment, and recovery.

The present study explores and illuminates the experiences and perspectives of older persons on ageing, alcohol use, treatment, and recovery from an alcohol problem, as well as their description of what constitutes healthy ageing. We examined these issues from the perspective of older persons in order to understand the challenges and strengths they face in relation to alcohol use and healthy ageing.

### Theoretical frameworks

In this study, we have taken an interpretative approach where we aimed to explore and illuminate the study participants’ subjective and narrative reality pertaining to their experiences of ageing, alcohol use, alcohol problems, treatment, and recovery. We also acknowledge that (a) we view addiction, recovery and ageing as multidimensional processes and (b) we believe treatment and care should address the biopsychosocial aspects of these processes and incorporate the individual’s needs, values, and experiences. As a result, two related approaches to health and illness, a person-centered care and the biopsychosocial model—both of which emphasize the centrality of the individual’s experiences— serve as interpretative frameworks for the study.

*Person-centered care* places emphasis on incorporation of the individual’s values, experience, preferences and expressed needs into the care process [35]. Person-centered alcohol treatment, hence, involves diversifying available treatment options, matching treatment and support services to the individual’s needs, and empowering the individual to participate in the decision-making process [36, 37].

The *biopsychosocial model (BPS)* of substance use disorders posits that alcohol problem prevention, assessment, treatment, and recovery should take into account the dynamic interactions between biological, psychological and social life domains of the individual. The BPS is also valuable for understanding the ageing process [38]. The biological perspective relates ageing to biological changes such as changes in physical appearance and the presence of diseases; the psychological perspective highlights that self-perception of ageing can affect the ageing process through preventive health behaviors, resilience and health service use [39, 40]; the social perspective includes environmental factors that can affect the ageing process, such as social networks, retirement, societal views on ageing, and the organization of welfare systems.

## Materials and methods

We used the Standards for Reporting Qualitative Research (SRQR) guideline for reporting of the procedures and findings of this study [41]. Detailed descriptions of the study setting, participant recruitment, data collection and interview guide are available at the Swedish National Data Service [42] and in Supplementary material 1.

### Participants and settings

Participants were treatment seekers purposefully recruited from a specialist outpatient alcohol treatment clinic staffed with physicians, nurses, psychologists, and other health workers with expertise in the treatment and management of substance use disorders. Patients attend the clinic through self-referral. In Sweden, substance use disorder treatment is publicly funded.

Eligible study participants were aged 55 years or more, spoke Swedish, experienced their alcohol drinking as problematic, and sought treatment for alcohol problems after the age of 50 years. By setting the cutoff age at 55 years, we hoped to capture key life events in the pre-retirement period.

Clinic staff approached potential participants, explained the study, and solicited study participation. Individuals who volunteered to participate provided a first name and telephone number (*n* = 13). The first author (WBJ) contacted potential participants, assessed eligibility, provided detailed study information, answered potential questions, and scheduled an interview. One person declined to participate and two did not respond to the investigator’s telephone call.

Study participants were sent informed consent materials with a Zoom link or telephone number for an interview. A reminder email and text message were sent 48 h prior to the interview. Participants had no need for technical assistance as they were familiar with Zoom due to having many of their treatment sessions online during the COVID-19 pandemic.

The audio recorded interview began with participants verbally confirming their consent to participate. Data analysis of the first eight interviews showed sufficient information power and no new categories emerged [43]. This adequacy was due to the purposive sampling method, quality and depth of the interviews, and analysis strategy [43, 44]. Therefore, no additional interviews were conducted after the tenth participant. The final sample included eight men and two women aged between 61 and 73 years who lived independently in their communities.

### Data collection

In-depth interviews used a semi-structured interview guide organized with three domains related to the study objective: a) Getting older; b) Describing alcohol use and treatment; c) Linking ageing, alcohol use and treatment. At the end of each interview, we obtained information on age, gender, civil status, education, main source of income in the past 12 months, household income’s ability to cover living costs, perception of current health, and perception of current health compared to others of the same age (Table 1).

**Table 1:** Characteristics of study participants and interview length

	Frequency
*Age*	
55–59 years	-
60–64 years	4
65 years or older	6
*Gender*	
Woman	2
Man	8
Other	-
*Current Civil status*	
Married or cohabiting	7
Separated or divorced	3
In a relationship but not living together	-
Single	-
*Education*	
Primary education	2
Secondary education	3
University or other higher education	5
*Main source of income in the past 12 months*	
Fulltime employment	3
Regular parttime employment	1
Irregular parttime employment	-
Sickness or disability allowance	-
Unemployed	-
Retirement pension	6
*Household income’s ability to cover living cost*	
Very easy	7
Easy	1
Somewhat easy	1
Somewhat difficult	1
Difficult	-
Very difficult	-
*Perception of current health condition*	
Very good	6
Good	3
Fair enough	-
Somewhat bad	-
Bad	1
*Perception of current health condition compared to others in my age*	
Very good for my age	4
Good for my age	4
Fair enough for my age	1
Somewhat bad for my age	1
Bad for my age	-
*Interview length*	
Below 60 min	-
60–74 min	1
75–90 min	2
91–128 min	7

Interviews were completed online between December 2021 and April 2022 due to COVID-19 travel restrictions imposed and required 74 to 128 min [mean (SD) = 100.5 (18.4); median = 103 min] excluding breaks. The audio conversations were recorded with an external recorder. Two investigators conducted each interview. WBJ participated in all interviews, ME in four and FS in six interviews. The three interviewers had no pre-existing relationships with participants and were not involved in the treatment provided at the clinic. The fourth author (AH) contributed to the recruitment design and training of recruiting staff.

Two trained undergraduate social work students transcribed interviews verbatim after signing on data integrity. Files were password protected and transferred through an encrypted file transfer system. WBJ checked transcribed text against the audio recordings.

### Data analysis

Qualitative content analysis guided the analysis [45, 46]. We decided to use qualitative content analysis as we aimed to explore and interpret the experiences and perspectives of older persons on the interplay between alcohol and ageing. Interview texts were read in their entirety several times to establish a sense of the whole. Three content areas were identified. Description of: a) ageing and healthy ageing; b) alcohol use and its relationship with healthy ageing; c) resources related to ageing and alcohol consumption reduction. The main coder (WBJ) divided the text in each content area into meaning units according to their content and context, condensed and coded each meaning unit related to the study aim. All codes were compared, and similar codes were grouped into clusters and labelled to form sub-categories (Table 2). Related sub-categories were then sorted and abstracted into categories. The analysis until the formulation of manifest content (subcategories and categories) was facilitated by NVivo version 1.7 [47]. Finally, the underlying meaning (latent content) of the categories was formulated into themes. Even though the analysis process was inductive, it involved several comparisons between the whole and parts of the text to confirm the validity of codes, subcategories, categories, and themes. The analysts (WBJ, FS and ME) held iterative meetings to reflect on decontextualization (condensation and coding) and re-contextualisation (sorting, abstraction and interpretation) of data, categorical decision and theme formulation and preliminary results validation. In the final step, WBJ and AH met to review the categories and themes, strengthening the credibility of the analysis.

**Table 2:** Example of condensation and abstraction process

Meaning unit	Condensation	Abstraction	
		Code	Sub-Category
As I said, you start to realize that there may not be more than 15 years left, maybe only 10 years left that you can be active.	Realizing that one might have only 10 or 15 years to stay active	Realizing the uncertainty how long one can stay active	Being uncertain about the future
the closer you are the more judgmental you are. I’ve chosen not to talk about it [drinking] with my closest ones because I don’t know if it would help anything, or if it would be upsetting	The closer you are the more judgmental you are, so I have not told my close friends about my alcohol problem	Not telling friends about alcohol problem due to fear of being judged	Having difficulty to disclose alcohol problem to close ones due to stigma
…I see it this way that… as it is now, I have a very free life because I can choose for myself whether I want to work or not	Having free life because of I can choose myself whether I want to work or not	Having the freedom to choose continuing working or not	Freedom to act according to one’s own terms
I have been fulfilling my social responsibility my whole life, since I was 18 years old. Working day and night. Now it feels great to be able to say no to that [others’ demands]. As an older person you own your time… it’s great that I own my time…I decide	After long period of working day and night, it feels good to say no and to own your time as an older person and decide for yourself	Being able to decide how to stir one’s time	

### Ethical consideration

Ethical approval for this study was obtained in advance from the Swedish Ethical Review Authority (Dnr 2021–02240). Informed consent was obtained orally from the participants and recorded. The study was performed in accordance with the Helsinki Declaration and the Belmont Report. All data were collected and processed in accordance with the General Data Protection Regulation (GDPR) and after notification of personal data processing at Umeå University (PUR 2021/99). Participants were not provided compensation for study participation.

## Results

The analysis identified three themes and nine categories (Table 3).

**Table 3:** Categories and themes revealed during the analysis

Categories	Themes
Living day by day with losses that creep up with ageing	“**Tipping the balance**”
Leveraging newly found freedom, accumulated experience and wisdom	
Understanding what healthy ageing entails and devising strategies to age well	
Living with hidden alcohol problem for long time	“**Staying behind a veil**”
Ambivalence towards alcohol’s effect on ageing	
Admitting one’s own alcohol consumption as problematic and deciding to seek treatment	“**Lifting the veil**”
Getting the right support to set personal goals and resolve alcohol problem	
Adopting strategies to reduce alcohol consumption	
Experiencing positive changes and hopefulness despite occasional return to heavy drinking	

### Theme 1. “Tipping the balance”

The first theme covers three categories describing participants’ experiences of living with age-related changes (both gains and losses), their understanding of what healthy ageing entails, and the strategies they devised to maintain important resources in order to positively affect their own ageing trajectory as they become older.

#### Category 1.1. Living day by day with losses that creep up with ageing

Participants identified a decline of resources or losses related to ageing as negative changes they wanted to delay or mitigate. The negative changes included a gradual decline in physical and mental capacity and health conditions (e.g., cardiovascular diseases, cataracts, neck, back and knee pain). Participants noted these changes in their daily activities which were becoming more difficult. One participant said *‘…I usually sit and play with my grandchild on the floor. Then when I have to get up, I notice that I am not so young anymore.’* (P2), while another participant observed *‘Being old means, I notice I forget more’ (P10).*

Shrinking social network was another negative change participants experienced with ageing. They feared that their network would decline further due to their own ageing, others getting older and dying, and the difficulty of building new friendships. A participant reflected *‘…Most of the people I know, they lie now in the cemetery. I know more dead people than alive at this point…’* (P5).

When asked about the negative changes, participants, including those who retired before the age of 65 years, raised the topic of retirement. They explained that retirement reduced social contact, precipitated a loss of engagement and they felt invisible. A participant complained *‘…you don’t feel that you are needed, and it’s quite a scary feeling… that from the email being full every day… to only receive advertising mail today…’ (P8)*. Those who still worked were aware of the risk of losing the stimulation and social contact their work offers and feared a monotonous post-retirement life.

Participants observed they avoided long-term planning and made decisions for the present and the near future because of uncertainty about the future. Uncertainties included the participants’ ability to stay physically active and independent in the future. One respondent asserted *‘…you start to realize that there may not be more than 15 years left, maybe only 10 years left that you can be active…’* (P1).

#### Category 1.2. Leveraging newly found freedom, accumulated experience and wisdom

Despite the stated losses with ageing, interviewees reflected how experience helped them deal confidently with daily life circumstances. They felt more tolerant and secure. One participant noted *‘Even if you lose some of your physical and cognitive skills, with experience you feel more secure’* (P7). Respondents, moreover, felt the experience and wisdom they acquired helped them avoid mistakes and provide proven solutions in work-related tasks.

Another positive change was the availability of time following retirement and more opportunity to engage in valued activities – exercise, travel, visiting relatives and childhood friends, home renovation, and involvement in local politics. They appreciated having freedom to act according to their own terms after years of fulfilling family and work-related demands. The participants also expressed a sense of urgency. They were aware that they had limited time to live with such freedom and capacity. According to a participant *‘… If there is something which gives me satisfaction, it is that I can steer my time…the most important thing I have NOW is time, it is my most important asset that I know. I want to use it wisely…’* (P3).

#### Category 1.3. Understanding what healthy ageing entails and devising strategies to age well

Respondents described ageing as a mixture of gains and losses. They expected that, at certain time points, the losses would outweigh the gains *‘… you will become old, and life will shrink somewhere soon…’* (P8). They, however, recognized the variation in resources individuals have access to affects their ability to prevent the decline of their quality of life as they age. When discussing resource variation, participants compared themselves to their parents, partners, and friends. They viewed healthy ageing as having the capacity to tip the balance by delaying as many losses as possible and continuing to engage in meaningful activities, a process which required their agency. Most highlighted the importance of maintaining their health and avoiding injury. Health maintenance required changing alcohol use *(we will discuss this in detail later)*, disengaging from physically demanding activities, adjusting the type and intensity of activities to current resources *‘… I always compensate for the gradual physical decline with practice… When you are older, you become slower. Then, you need to compensate with wisdom, I use my head more to spend my energy the right way instead of galloping all the way…’* (P7).

Engaging in physical activities which provided continuity in the face of declining resources was also important for participants as they age. One participant, for example, saw playing golf as instrumental in staying active after he stopped playing football and badminton in middle age, and during the COVID-19 pandemic when socialization was limited. The participant hoped golf would provide continuity in the future *‘…I think I will be able to play golf until I can’t sit by myself…’* (P1).

Healthy ageing was not equated with being disease-free. When talking about maintaining good health, participants recognized the inevitability of medical conditions as a normal part of ageing. However, they also emphasized the importance of preserving cognitive skills and mobility for as long as possible, as these are necessary resources for pursuing meaningful activities and maintaining autonomy, dignity, and independence. Interviewees, for example, talked about ‘*trying to keep going as far as possible*’, *‘eating well and exercising in order not to get old too fast’*, *‘wishing to maintain somewhat active life until 81 years’* and shared their worry of *‘being a burden on family’* and *‘not making others happy’.*

Maintaining family relationships, social networks and community engagement was important for participants’ healthy ageing. A participant commented that *‘…[about ageing well], it is necessary that … you have a relationship…//… that you have close relationships all around you, that you have an intellectual exchange…’* (P8). Even if participants reported experiencing social exclusion and disengagement as they became older, they discussed strategies to mitigate the loss. Some used the free time and independence that comes with retirement to rekindle childhood friendships, look after grandchildren, improve relationships with partners and engage in meaningful activities such as walking out in the nature, traveling, and sailing. Others sought to meet new people and build friendships through golf club membership, skiing and other physical activities. Participants who continued to work used their employment to maintain social relationships.

Another resource important for healthy ageing was staying curious about life and pursuing stimulating and meaningful activities even when retirement removes opportunities the come with labor participation. A participant said *‘…I think part of ageing well is continuing to be curious about life. Then, I think you age well, then I think life is worth living all the time…’* (P10). Participants often spoke about the importance of maintaining other resources, such as mobility and cognitive skills, to stay curious about life. They observed how the COVID-19 pandemic interrupted meaningful activities such as travel and social gatherings and were looking forward to visiting new places and learning new cultures.

Participants related having financial security with healthy ageing as it enabled them to maintain their autonomy and ability to engage in stimulating activities. Working beyond retirement age due to financial instability was considered an example of not ageing well.

According to respondents healthy ageing entailed having a home where they could age in place. Many had recently moved to a new place or planned to do so because they wanted to adjust to their current and expected physical and financial capacity. They hoped to stay in their home for as long as possible and purchase home-care services when needed instead of living in an old-age care facility. Home is something beyond a shelter and one respondent explained *‘…a home is not only four walls…’* (P4), they wanted it to be a place they spend good time with family and friends.

In brief, participants actively tried to minimize or delay age-related losses and leveraged the gains that came with age. Despite perceived age-related losses, they often talked about what was good with their ageing and often compared their ageing with others in their surroundings. They were, however, uncertain about the future. They wanted to do more to maintain the resources they needed for healthy ageing, including moderating their drinking.

### Theme 2. “Staying behind a veil”

The second theme covers two categories encompassing participants’ experiences of living with a hidden alcohol problem and having ambivalence towards alcohol’s impact on their ageing.

#### Category 2.1. Living with a hidden alcohol problem for a long time

Participants described alcohol’s role in their lives before drinking became problematic. Alcohol was part of socialization with friends and relatives and provided a means to maintain social networking, an important resource for healthy ageing. Alcohol was readily available in their surroundings and some felt pressure to drink even when they did not want to drink *‘…you become a little weird if you don’t drink…people ask “why don’t you drink? Are you sick or what?”…’* (P6). Many participants discussed how drinking was integral to their work context in business deals and work retreats or as a means of relaxing with colleagues after work. Alcohol was *‘a means to enjoy life’, ‘quality of life’* and *‘something good to have’*. According to one respondent *‘… in some way… [alcohol] fits to the overall picture of ageing well. [Ageing well] is not just about taking care of your body, it's also about allowing yourself to enjoy the things around you…//…In that sense, wine and other alcohol drink are part of it. [Alcohol] is such an important part that I am not prepared to give it up…’* (P8). Alcohol functioned as a reward for a completed task, a conclusion to a work week, taking care of a garden or exercising.

The experiences related to drinking shifted from fostering socialization and good quality of life to it occupying daily life and taking over a large part of their time. Some described key events in their life associated with the onset of their increased alcohol consumption: an unexpected loss of partner, a divorce and a sudden loss of a social network, financial bankruptcy, and medical problems. Others stated that their alcohol consumption increased gradually from occasional or weekend drinking to drinking more, and more often. One event associated with increased drinking was retirement. More time became available, and for some life became slower-paced and monotonous. The structure required during work was removed. Some drank on more days during the week. Others drank more per day. An interviewee remembered *‘…I might drink from the afternoon until eight in the evening, then I don’t drink anymore… it's spread across 5 h…then it means I drink a bottle…//…When I sat in [my office], things went well during the day, then I couldn’t do anything. But now I can drink during lunch and when I get home…’* (P2).

Participants used alcohol to inhibit anxiety and feeling of loneliness, and to cope with difficult situations *‘…I have used [alcohol] to escape… it could have been that I worked too much, or I had a problem, often relationship problems, of course…, well, I go out and have a few beers instead, then I feel like I am relaxing. But what I do, I think, is that I put something difficult [feeling] aside and have a beer instead…’* (P3).

Participants reflected on situations that made them realize that they had alcohol problems. For some participants, alcohol resulted in family conflicts. Others worried about their consumption when they were drinking again despite physician advice to reduce their drinking and despite feeling regret, anger and worry about their health. Participants realized their drinking was problematic when they noticed that they had strong cravings for alcohol, struggled to abstain even for a couple of days and that alcohol drinking was taking over other activities.

Participants often avoided seeking treatment because they feared they would be instructed to abstain from alcohol, an outcome which did not match their wish of reducing their drinking. Many had attempted to reduce their drinking level but found it difficult to change. When explaining their unsuccessful effort and worsening of the situation, participants used phrases such as ‘*feeling powerless’, ‘being stuck in a situation that I don’t want to identify with’, ‘never thought I would fall this deep’,* and *‘not feeling the effect of alcohol as before despite drinking more’*. Participants did not like that they increasingly ‘*sought comfort in a bottle*’.

Once alcohol became a problem which could not be easily resolved, participants experienced shame and guilt. They avoided disclosing the problem to their close ones because they were afraid of facing stigma and losing their social network. A participant said: *‘…the closer you are the more judgmental you are…, I've chosen not to talk about it [drinking] with my closest ones…’* (P7). Alcohol problems were not visible in other contexts such as workplace because participants drank more at home or outside the work hours and because they often fulfilled their responsibilities. They were able to control their drinking level when socializing with colleagues and friends. Participants managed their relationships by drinking less when with their family and friends. Once family members noticed the problem, they expressed their disapproval which increased feelings of shame and hesitancy to seek help as *‘…I noticed that it is not good if my spouse goes and points the finger every time [my spouse] thinks I drink too much. Because in some way it even becomes counterproductive… that it's okay to drink as long as [my spouse] doesn't see, or notice…’* (P9). Other participants stated that it took them too long to admit to family members that they had an alcohol problem and when they did, their family reacted with grief and anger. A participant elaborated, *‘…. shame and guilt made me not seek help earlier….after many years, I laid my cards open and told my children that I had an alcohol problem… They cried…they could not accept their [parent] had such problem… I experienced they felt contempt for me … the image they had about me was gone…’* (P5). In summary, participants initially viewed alcohol as integral to social life and enjoyment, but gradually experienced the negative consequences of heavy drinking.

#### Category 2.2. Ambivalence towards alcohol’s effect on ageing

Although participants worried about the amount and frequency of their drinking and strained relationships with family, they lived with an alcohol problem for long time. Participants described the stigma, loss of control, dependency, and a perceived lack of alternatives to abstinence-based treatments that inhibited seeking treatment. They also discussed their previous ambivalence towards alcohol’s effect on their resources for healthy ageing and reasons for the indifference.

First, many participants doubted alcohol was affecting their health; they maintained good physical and cognitive functioning and considered themselves healthier than their peers who did not have an alcohol problem. Some felt they were too old and that it was not worth the effort to reduce their drinking for the sake of longevity *‘…I understand alcohol is not good for the body… I thought “I am too old, and it did not matter if I stop drinking and live 2 years longer or die 2 years earlier”….Should I live a boring life just to live 2 years longer?…’* (P2). Although doctors advised some to reduce their drinking because liver function tests were elevated, they returned to drinking when a follow-up test showed improved liver function.

Second, even if their relationships with partners and children were increasingly difficult, the relationships were either not *‘that bad’* or that they did not *‘have a carrot and stick’* to motivate reduced drinking. Many participants married and were able to be with their children and grandchildren as long as they managed their drinking during those occasions.

Third, participants had financial security and housing. Alcohol had not affected their finances they were not *‘bankrupt’* or *‘looked worn out’*. In summary, respondents believed for long time that they were able to manage the important resources as they aged and that their alcohol use was not of concern. It was when they perceived the increasing effect of alcohol as unsustainable and an immediate threat to the future status of their resources that they decided to make change.

### Theme 3. “Lifting the veil”

The third theme’s four categories describe how participants, after living with problematic alcohol use, overcame stigma and ambivalence, admitted their problem use, sought treatment and identified their treatment goals. The categories describe the treatment the participants received, the strategies they adopted to meet their treatment goals, the positive changes they experienced and how those changes helped them stay hopeful even when they had a recurrence of heavy drinking.

#### Category 3.1. Admitting one’s own alcohol consumption as problematic and deciding to seek treatment

Despite stating that they currently have the resources they need for healthy ageing, participants were worried that their alcohol use had become problematic. When asked what contributed to their decision to seek treatment, participants reported that admitting their alcohol problem to themselves and others was a crucial step toward helping them seek treatment. They described their previous drinking as *‘not sustainable anymore’*, *‘too much’*, *‘cannot keep like this anymore’*, *‘alcohol deprived me of myself’, ‘have had other life goals than being alcoholic’* and *‘ageing with alcohol problem is not what I envision’.* They worried that if alcohol had not affected their ageing it would start to do so.

Health was one resource their alcohol use threatened. A recently hospitalized participant who had a discussion with his specialist doctor admitted: *‘alcohol might have contributed to my [medical condition]…’ (P9).* Another participant worried his past drinking damaged his cognitive skills when he could not solve sudoku, a game he plays to maintain his cognitive functioning.

Others realized the extent of their alcohol problem and its effect on their relationships when they began to hide drinking from their partners or when they drifted away from their partners. Some decided to modify their drinking because they were afraid an alcohol problem would inhibit engagement in their profession, family, and community. A participant, for example, stated the need to make an immediate change because of a new role as grandparent which required active participation. Another spoke of his disappointment when he lost his professional driving license due to an alcohol problem and he needed to make a change to be able to get it back. In summary, many participants recognized the urgent need to reduce their alcohol consumption when the consequences of their drinking became tangible and they understood the possibility of losing resources needed for healthy ageing.

#### Category 3.2. Getting the right support to set personal goals and resolve alcohol problems

Participants confided that they did not want to quit drinking. Their goal was to maintain lower risk consumption and continue *‘enjoying a beer or a glass of wine with dinner’* or *‘drinking during weekends and holidays.’* As a result, they avoided abstinence-based treatments. They had not received alcohol treatment from social services either because they had stable financial and housing conditions. As a group, however, they had frequent medical comorbidities and accessed healthcare services. Several noted that their trusted general practitioner or psychiatrist recommended they seek care at the specialist outpatient alcohol treatment clinic. The recommendation motivated initiating contact with the clinic and booking an appointment.

The respondents appreciated that clinicians at the clinic asked them to identify personal recovery goals and that they were able to negotiate achievable goals and recovery strategies. They continued their treatment because the clinicians respected their goal setting autonomy, trusted their capacity to implement negotiated strategies and provided guidance instead of directives. A participant said, *‘…the therapy itself doesn't instruct you how much exactly you should drink. I mean, their way of putting it up is different. They ask you, what is your goal and then they try to help you with motivational ideas…’ (P4)*.

Establishing a working therapeutic partnership with care providers was important for the participants. They mentioned that building trust and having an open dialogue with clinicians required time and depended on the partnership dynamics. Respondents appreciated the clinicians’ non-confrontational and gentle motivational approach. The patient/clinician partnership was a key ingredient for participants’ recovery *‘…I finally got in touch with [psychologist] who guided me well through this period…//…we talk about everything possible…[the psychologist] is so supportive and understanding…is not judgmental but can also ask those tricky questions that I have to start thinking about… we have had a long-term contact and the relationship has become very meaningful for me…[the psychologist] is the right person in the right place’* (P5).

In addition to incorporating participants’ autonomy in setting goals and negotiating strategies, participants stated that clinicians provided them with feedback during treatment which strengthened their agency to reduce their alcohol consumption. Feedback included information about health risks associated with high alcohol consumption, interpretation of initial and follow up liver function test results, and reflection on strategies participants applied to achieve set goals.

#### Category 3.3. Adopting strategies to reduce alcohol consumption

Having support and treatment that aligned with their life goals, enabled participants to try different alcohol reduction strategies. Many of the strategies the participants adopted encompassed the resources they identified as important for healthy ageing. Some involved their family to help achieve their drinking modifications. A participant, recalled the strategy he negotiated with the therapist *‘….[when the therapist asked about my goals]…//… I said, “Well then we can set as a first goal that alcohol is allowed on Friday Saturday and Sunday' and, of course, not to drink alone. Then I drink with my partner and then my partner keeps track of the levels”…’* (P8). Others engaged in alternative activities such as taking up interests from earlier in life, exercising more during weekdays, engaging in excursions with their partners and engaging in community.

Participants reported that the conversations with the clinician helped them to identify thoughts and feelings that often led to drinking excessively. They focused on increasing their internal resources such as coping resources and seeking therapy for stress management, trauma, depression and anxiety.

Other strategies included recording their daily consumption using a calendar, avoiding drinking situations (e.g., staying up late night alone, visiting city center during the day), substituting non-alcoholic drinks for alcohol during socialization and replacing distilled spirits with low-alcohol beer.

#### Category 3.4. Experiencing positive changes and hopefulness despite occasional return to heavy drinking

The positive change since study participants entered alcohol treatment was not limited to less drinking. Many perceived improvements in multiple life domains; improved physical and mental health, strengthened family relationships, less spending on alcohol —domains participants associated with healthy ageing. The improvements encouraged them to continue their *‘daily battle with alcohol’.* A participant who noticed these changes and his newly found strength to resist stimuli explained, *‘…[since drinking less] I feel healthy, much healthier surely… I have a better mood, the best it has been. My relationship is great, I have much better patience with things. I feel great physically and mentally., it's just win–win situation, what I really forget is also the financial factor as well, there's a lot of changes… There are no negative things…//… I want [my relatives] to live their life and if we have a party at home, I can drink my non-alcoholic bubbly and they drink their alcohol…’* (P7).

Some respondents, experiencing positive change since accessing treatment reported occasional setbacks (drinking excessively) while others described their recovery process as sometimes being challenging and needing their active effort which can be exhausting. A participant who exercised on weekdays and only drank on weekends observed *‘… I drink significantly less, I don’t drink as often. I don’t feel the same pleasure from alcohol anymore. If I drink too much, I get really bad the next day. That means I lose a day there… so no, I consciously try to limit my drinking. It can be exhausting at times but of course I feel good about it… sometimes you have to fight with it, so to speak.’* [P6].

As recovery journeys continued, respondents were hopeful of strengthening their capacity to moderate drinking and maintain their healthy ageing resources. One explained, *‘… I’m satisfied. I’m happy and I feel “yes! how nice… God! how good!” I feel satisfied with myself and think that now it’s going to work out, now it’s going well…then I feel both pride and joy because that’s who I want to be…’* (P5).

## Discussion

In this study, older persons who sought treatment for problematic alcohol use were interviewed to explore their experiences of ageing, alcohol use and treatment for an alcohol problem. Participants reported both positive and negative experiences with their ageing and their alcohol use. They were, however, active agents shaping their own ageing trajectory in the face of age-related resource decline. Furthermore, participants overcame stigma and ambivalence to receive help in order to realize their recovery goals after living with alcohol problems for a long time.

### Ageing and healthy ageing

Participants experienced ageing as a process with negative (losses) and positive (gains) changes. Age-related losses included declines in physical and cognitive capacity, a shrinking social network, declining stimulation and increasing future uncertainty. Age-related positive change (gains), on the other hand, included accumulated experience and wisdom, increased patience and tolerance, improved self-confidence and sense of security, autonomy, and availability of free time. Our study participants were older persons with lived experiences of alcohol problems and recovery. Other studies on older persons with different characteristics have reported ageing is experienced as a multidimensional process with positive and negative changes [48–51]. The present study suggests that participants understood healthy ageing as a process that is characterized by their active role in *expanding, maintaining* or *adjusting to* the resources they have in order to lead active and meaningful life while preserving their autonomy, dignity and independence for as long as is possible. Reported critical resources include physical health, cognitive capacity, emotional wellbeing, social network and community engagement, family relationship, curiosity, and financial and housing security. Accordingly, healthy ageing is understood by participants as a personal, multidimensional, and time-constrained process which encompasses the biopsychosocial life domain of the individual and involves the individual’s constant negotiation with and adaptation to their environment instead of a mere absence of diseases.

Despite living with somatic or mental health conditions, many study participants evaluated their health as very good or good in general and compared to their peers. They reported tapping into age-related gains (e.g., wisdom and experience) to compensate for declining ability (e.g., physical capacity). Previous studies have found that older persons have high adaptive capacity to adjust to age-related changes and manage their life by resetting goals, adjusting their activities to their reduced capacity, and utilizing alternative strategies and new means [10, 49, 52].

Both retired and working participants understood ageing and related changes through the lens of retirement. A shrinking social network and less stimulation were linked to a loss of social contacts and disappearance of work-related demands that follow exit from workforce. On the other hand, retirement contributed to the age-related gains by providing increased freedom to act on personal terms (autonomy) and more time to engage in meaningful activities. Retirement was, thus, a period of change that resulted in realignment of social network structure and adjustment to a new context. Like the current study, others have shown that those who continued to work beyond retirement age often did so to avoid the negative changes that come with retirement [53–55].

### Alcohol use and alcohol problem

Our study participants described alcohol use as a means of socialization, enjoyment of life, managing stressful situations, and relaxation after completion of a task. They felt drinking contributed to their healthy ageing by facilitating emotional wellbeing and social connectedness. Similar reasons for alcohol use were also reported by older persons in the UK, New Zealand, Finland and Denmark [56–63]. However, these earlier studies did not investigate how older people described the interplay between their ageing process and alcohol use, a gap which the present study aimed to address.

Perceiving alcohol use as a part of healthy ageing could explain why the study participants lived for a long time with an alcohol problem, even as they became aware that drinking was eroding the same resources they regarded as important for healthy ageing. Many participants tried to reduce their drinking but were unable to and sought treatment only when they perceived alcohol as an immediate threat to their healthy ageing. Cognitive appraisal of the problem and perceived threats to physical and mental health, social networks and engagement, autonomy, and independence have been identified as important treatment facilitators [64, 65].

Even when individuals acknowledge their alcohol problem, they often underestimate the risk of alcohol use and problem severity [57, 66, 67]. Moreover, only a minority of individuals with an alcohol problem access treatment, often with a lag of eight to fourteen years between problem onset and treatment entry [25, 68–70]. This study identifies ambivalence, stigma, dependency, and the lack of treatment options as barriers contributing to this treatment gap, corroborating previous research findings [29, 70–73].

### Alcohol treatment and recovery

In this study, participants preferred supportive and nonconfrontational alcohol interventions that focused on improving their coping and social skills. They appreciated the trust-based therapeutic relationship with clinicians, which was motivational and reinforced their autonomy and agency through guidance and feedback. Many of the negotiated strategies for reducing alcohol use encompassed the internal and external resources for healthy ageing which were identified during our interview. Furthermore, participants responded to intervention approaches which elicited their concern, incorporated their treatment and life goals, and considered them partners in treatment planning. The importance of patient participation and therapeutic alliance in person-centered care delivery and treatment success has also been highlighted in previous studies [9, 36, 64, 74, 75].

Understanding a patient’s important resources for healthy ageing can inform alcohol treatment and recovery. Our study participants perceived alcohol recovery as akin to the process of healthy ageing, emphasizing its personal and multidimensional nature, where their active involvement is required to achieve favorable outcomes. They also described how regaining their agency and improving their resources for healthy ageing catalyzed a continuing recovery process. This view is in line with recent conceptualization of recovery as a dynamic process characterized by improvements in biopsychosocial functioning, quality and purpose of life [34, 76–78].

Our study participants acknowledged the ongoing effort required to maintain their recovery, aligning with findings from previous reports [31, 76, 79]. Some participants experienced occasional returns to heavy drinking during their recovery journey. Despite these setbacks, they strived to sustaining moderate drinking as they continued their recovery process. A growing body of literature supports that moderate drinking can be a viable pathway to resolution of an alcohol problem [30, 80–84]. While abstinence is a safer recovery route for many individuals due to the negative consequences associated with alcohol consumption, those who occasionally engage in heavy drinking but maintain high biopsychosocial functioning after treatment should also be considered in recovery [77, 81, 84, 85].

### Implication

One of the key areas for healthy ageing identified by the World Health Organization (WHO) is the alignment of health systems with the needs of older persons they serve [1]. Our analysis suggests older persons may seek alcohol treatments and interventions which incorporate their treatment and life goals with respect and emphasis on autonomy and personal agency. Eliciting and understanding older persons’ descriptions of resources for healthy ageing and their interaction with alcohol use and alcohol problems can inform clinicians when promoting healthy lifestyle and identifying domains of recovery capital that present risks and strength for service users.

WHO’s key areas for action on healthy ageing also include improvement of measurement, monitoring and understanding of healthy ageing [1]. Including older persons’ perception of healthy ageing is instrumental in testing the validity of existing healthy ageing models and indicators. Empowering partnerships can facilitate the development of interventions that are acceptable for older persons. In this study, for example, older persons did not associate abstinence from alcohol with healthy ageing. For some older persons, an abstinence requirement may not be acceptable and could inhibit health service utilization. Therefore, expanding alcohol interventions to include moderate drinking as a treatment goal may encourage earlier treatment seeking, reduce alcohol-related functional impairment and improve biopsychosocial functioning among older people.

In Sweden, addiction treatment has traditionally been provided by social services which primarily serve individuals with social problems such as unstable finance and housing. Many older people with alcohol problems, however, have stable psychosocial functioning and do not access social services. A population-based survey found that only five percent preferred to receive treatments from social services while almost 90% preferred healthcare services [71]. Given that many older persons receive primary and specialist care services, training healthcare professionals such as general practitioners and nurses on ageing and addiction can help to identify alcohol problems early and provide age-sensitive treatment for older persons. Additionally, the inclusion of allied health workers such as social workers and occupational therapists, who are trained on ageing and addiction, within both primary and secondary healthcare services can provide an additional point of contact for delivering integrated care to older persons with alcohol and related problems.

### Methodological consideration

Study participants were self-selected as they were purposefully recruited from a specialist outpatient alcohol treatment clinic and attended the clinic through self-referral. The sampling method was appropriate because we sought participants who were able and willing to give us rich information on our research domains, healthy ageing and alcohol problems. Even if the participants varied in age, education, civil status, retirement status, comorbidities, alcohol trajectories, drinking pattern and context, most had better biopsychosocial functioning compared to treatment seekers in other studies [6].

The proportion of women interviewed for the study (20%) was lower than the proportion of women accessing treatment at the recruitment clinic (40%) during the study period. As the study did not aim to explore gender-specific characteristics of alcohol use and ageing, we opted not to increase the number of women in the sample. Nonetheless, we acknowledge that we might have discovered gender-specific manifest content (subcategories and categories) had we interviewed more women.

Online interviews present some challenges, such as limited ability to observe nonverbal communication, variations in call quality and access to the internet [86]. Participants in this study were given the option of conducting video calls with their cameras enabled, with an explanation of how this could assist us in understanding nonverbal cues. Many participants, however, opted not to turn on their camera, while the interviewers had their cameras turned on for all interviews. Two participants joined the Zoom interview by phone, while others used their computer or tablet. All participants were familiar with Zoom, having used the platform for their treatment sessions during the COVID-19 pandemic, and thus did not require technical assistance.

The study could have benefited from involving peer researchers and/or performing member checks for verification of the overall results. Despite this limitation, we took other measures to ensure the trustworthiness of the study. To safeguard the credibility of the study we collected rich data through in-depth interviews, held several meetings to confirm the validity of codes, subcategories, categories and themes, and used the full transcripts as units of analysis to preserve the context of meaning units during the condensation and coding phases. We formulated the questions in the interview guide carefully in order to avoid our preconceptions of the studied topics influencing the participants narration of their perspective. We did not provide the study participants with the existing definitions of addiction, recovery and healthy ageing and these definitions were not presumed within the analysis step. The manuscript includes representative quotations and illustrates the decontextualization and abstraction process. To address the dependability of the study, we followed a semi-structured interview guide, collected data within a relatively short time and provided a detailed description of the recruitment, data collection and analysis procedures.

The transferability of the study might be limited because participants had moderate drinking as a treatment goal, had good biopsychosocial functioning and were 61–73 years old living in a highly developed welfare state. Future studies can explore the same topic by including structural components which impact availability and access to psychosocial resources. Including population subgroups (e.g., those in ‘the fourth age’, those in natural recovery, accessed recovery support groups and/or abstinence-based treatments and those with socioeconomic disadvantage) can be helpful to assess the heterogeneity of older persons’ perception of healthy ageing, alcohol problem and recovery. Further research can also quantitively examine the interaction of age-related changes, resources for healthy ageing and recovery.

## Conclusion

This study suggests that older persons who are in recovery from problematic alcohol use describe their ageing as a mixture of gain and loss of resources. Healthy ageing and recovery from an alcohol problem are viewed by older persons as personal, multidimensional and interacting processes which entail their agency to tip the balance to favorable biopsychosocial functioning.

Older persons actively devise strategies to expand, maintain or adjust to the available internal and external resources to shape their ageing trajectory. This study suggests that the resources for healthy ageing interact with alcohol use, alcohol problems, and treatment and serve as capital for ongoing recovery. Older persons are more likely to respond to alcohol treatments which incorporate their treatment and life goals, appreciate their autonomy and agency, and consider them as partners. Therefore, treatment approaches that recognize older persons’ striving for healthy ageing and follow holistic approaches can be acceptable and effective for many treatment seekers.

### Supplementary Information

**Additional file 1.**

## Data Availability

Metadata description of the data and transcribed interview text are available at the Swedish National Data Service on https://doi.org/10.5878/j3hm-3w77, however, access to the data is restricted because of legal and ethical restrictions. Data access requests can be directed to Ellinor Gustafsson, administrator and directory coordinator at the Department of Social Work at Umeå University, Samhällsvetarhuset, plan 5, Biblioteksgränd 4, Umeå universitet, 901 87 Umeå, by clicking the “request data” button on https://doi.org/10.5878/j3hm-3w77at the Swedish National Data Service.
