# Supplementary material for: ‘Ageing with an alcohol problem is not what I envision’: reclaiming agency in shaping personal ageing trajectory and recovery from alcohol problems
Source: BMC Geriatr. 2023 Dec 16;23:866. doi: 10.1186/s12877-023-04573-y (PMC10724916; doi:10.1186/s12877-023-04573-y)
Supplement: Supplementary file 1 — Additional file 1. [file 12877_2023_4573_MOESM1_ESM.docx]

# Title: ‘Ageing with an alcohol problem is not what I envision’: Reclaiming agency in shaping personal ageing trajectory and recovery from alcohol problems

**Authors’ List, Order and Affiliation**

**Wossenseged Birhane Jemberie, MPH, PhD. ^1, 2, 3*^**

Postdoctoral Fellow. **1.** Department of Social Work, Umeå University, Umeå, Sweden;

**2.** Centre for Demography and Aging Research (CEDAR), Umeå University, Umeå, Sweden;

**3.** The Swedish National Graduate School for Competitive Science on Aging and Health (SWEAH), Faculty of Medicine, Lund University, Sweden.

E-mail: [wossenseged.jemberie@umu.se](mailto:wossenseged.jemberie@umu.se) ORCiD: 0000-0002-4378-6803

**Fredrik Snellman, PhD. ^1^**

Docent and Associate professor. **1.** Department of Social Work, Umeå University, Umeå, Sweden;

Email: [fredrik.snellman@umu.se](mailto:fredrik.snellman@umu.se) ORCiD: 0000-0003-3452-3953

**Malin Eriksson, PhD. ^1^**

Professor. **1.** Department of Social Work, Umeå University, Umeå, Sweden;

Email: [malin.eriksson@umu.se](mailto:malin.eriksson@umu.se) ORCiD: 0000-0003-0108-4237

**Anders Hammarberg, PhD. ^4^**

Associate professor. **4**. Centre for Psychiatry Research, Department of Clinical Neuroscience, Karolinska Institutet and Stockholm Health Care Services, Region Stockholm, Stockholm, Sweden;

Email: [anders.hammarberg@ki.se](mailto:anders.hammarberg@ki.se) ORCiD: 0000-0002-9976-5227

***Send correspondence to** Wossenseged Birhane Jemberie. Department of Social Work, Umeå University, 901 87 Umeå, Sweden. E-mail: [wossenseged.jemberie@umu.se](mailto:wossenseged.jemberie@umu.se)

**Running head: *AUD, recovery and healthy ageing***

# Supplementary Material 1

## **Setting and study participants**

Participants were treatment seekers purposefully recruited from a specialist outpatient alcohol treatment clinic, staffed with physicians, nurses, psychologists, and other allied health workers with expertise in the treatment and management of substance use disorders. Self-referred patients are offered a broad array of treatment options, including cognitive behavioural therapy, relapse prevention, 12-step facilitation therapy, motivational enhancement therapy and pharmacotherapy. Most patients (approximately 80%) choose moderation as their treatment goal and patients may change their goal to abstinence if treatment is not successful. Clinic records indicate that in 2021 the mean age of participants was 52.2 years and approximately 42% were women. Patients met a mean of 5.4 alcohol use disorder (AUD) symptoms; 48% of participants were classified as severely dependent (i.e., 6 or more AUD symptoms) according to the Diagnostic and Statistical Manual of Mental Disorders, Fifth Edition (DSM-5) (**American Psychiatric Association, 2022**). In general, patients have mild to moderate comorbid psychiatric problems.

The inclusion criteria outlined participants should be 55 years or older speaking Swedish and have experienced their alcohol drinking as problematic, have sought treatment for alcohol after the age of 50 years old. Participants with abstinence as a treatment goal and those who had a previous treatment history before age 50 were included in the study. A) Those who cannot or will not give informed consent and/or B) those who cannot/ will not participate in the interview through either zoom or skype or telephone were excluded from the study. The staff at the clinic initially approached potential participants and explained the aim of the study, soliciting their participation. Those who agreed to participate provided their first name and telephone number for further contact with the researchers. The first author (WBJ) contacted potential participants to further assess their eligibility, provide detailed information about the study, answer potential questions from the participants and set a date for the interview. One woman declined to participate in the study and refused to give a reason, while two individuals (one man and one woman) did not respond.

Eligible participants provided an address to be sent written study materials including information about the study, potential risks of participating and an email address for a psychologist in case they needed post-interview support. They were also provided with information on how their personal data would be processed and their rights according to the European Union's General Data Protection Regulation (GDPR). Participation in the study was voluntary, and participants were free to withdraw at any time without any consequences for their treatment and care. The written materials also included contact information for the researchers and a list of consent-related questions that would be asked during the agreed-upon interview date. Participants were emailed this written information along with a Zoom hyperlink or telephone number for the meeting. A reminder email and SMS were sent 48 hours before the interview. All participants were familiar with Zoom, as they had been using it for their treatment sessions during the COVID-19 pandemic and did not require technical assistance.

The audio recorded interview opened with a verbal confirmation of their consent to participate. Comprehensive data review and data analysis determined information power was achieved with the first eight interviews (i.e., no new categories emerged). Based on information power related to the purposive sampling method, the quality and depth of the interview and the analysis strategy no additional interviews were conducted after the tenth participant following previous methodological guidelines (**Guest et al, 2006; Malterud et al., 2016**). The final sample included 8 men and 2 women, aged between 61 and 73 years and living independently in their communities. Most of the participants in the study were married, had higher education, were retired, and had a very good financial situation. Nine out of the ten participants perceived their current health condition as good or very good, and eight participants perceived their health status as good or very good for their age.

## **Data collection**

We collected data through in-depth interviews using a semi-structured interview guide that was organized across three domains: a) Getting older; b) Describing alcohol use and treatment; c) Linking ageing, alcohol use, and treatment. We began the interviews by inviting participants to introduce themselves and transitioned to the topic domains. At the end of each interview, we gathered some background information from the participants, including their age, gender, civil status, education, main source of income in the past 12 months, household income's ability to cover living costs, perception of current health, and perception of current health compared to others who are the same age.

The interviews were conducted between December 2021 and April 2022 online due to travel restrictions put in place by the Swedish government to mitigate the spread of COVID-19. The interviews lasted between 74 and 127 minutes (mean (SD) in minutes = 100.5 (18.4); median = 103 minutes) excluding breaks. Only the audio files were recorded using an external recorder. Participants were given the option of having a video call with their camera on and were informed about the advantage of this in helping us understand nonverbal communication. Many participants chose not to turn on their camera, but the interviewers had their camera on for all interviews. Two participants joined the Zoom interview by phone, while others used their computer or tablet. Two investigators conducted each interview. The first author (WBJ) participated in all of the interviews, while ME participated in four interviews and FS participated in six interviews. WBJ (a man with a BSc in clinical dietetics and nutrition and a master's in public health) was a doctoral student at the department of social work. WBJ is currently a postdoctoral researcher at the same department. ME (a woman with a PhD in public health and a professor of social work) and FS (a man with a PhD in social work and a docent in social work) are experienced qualitative researchers. The three interviewers had no pre-existing relationships with the participants and were not involved in the treatment provided at the clinic. The fourth author (AH) is a man with a PhD in medicine, a docent in clinical addiction research, and a licensed psychotherapist. AH contributed to the recruitment design and trained the recruiting staff.

Table S1. Examples of questions extracted from the interview guide.

| 1. **On Getting Older** |
| --- |
| **A1**. What does getting older mean for you? |
| **A2**. What changes have you experienced as you get older?  *(Probing question: What changes do you think others can experience as they get older?)* |
| **A3**. What does aging well or healthy aging mean for you? |
| **A4**. What do you believe is important for you to maintain as you get older?  *Follow up : How do you feel about your ability to maintain these?* |
| **A5**. What do you think can contribute for someone not to age well (or have healthy aging)? |
| 1. **Alcohol Use and Treatment** |
| **B1**. How would you describe your relationship with alcohol?  *Follow up question: Have there been changes in the way you drink alcohol throughout the years? Please elaborate.*  *Follow up question: Can you tell us what led you to [increase/decrease/interrupt etc.]* drinking?*  *Note: *based on the answer for B1 and the previous follow up* |
| **B2**. For how long have you been drinking at a level you felt was problematic before seeking treatment?  *Follow up question: Have you tried to change your drinking before seeking treatment? How? If not, why?*  *Follow up question: Have you sought for alcohol treatment from social services or other treatment providers before contacting the specialist clinic?* |
| **B3**. What made you contact the specialist clinic for treatment?  *Follow up question: Was there any event or situation that led you to seek treatment?* |
| **B4**. How would you describe the treatment you received from the clinic?  *Follow up question: How do you feel about the treatment you received from the clinic* |
| B5. What changes have you noticed since you received treatment for alcohol from the clinic? |
| 1. **Linking Ageing, Alcohol Use and Treatment** |
| **C1**. How do you think alcohol is related with aging process? |
| **C2**. How do you think alcohol is related with aging well (healthy aging)? |
| **C3**. What do you feel would change in your ability to maintain what is important for you as you get older if an alcohol problem was not in the picture? |
| **C4**. What has changed in your ability to maintain what is important for you as you get older since you reduced your alcohol consumption? |

The interviews were transcribed verbatim by two undergraduate social work students. Following hands-on training from the first author on how to ensure data integrity according to GDPR during the work cycle, the students signed a data integrity agreement and received encrypted audio files. All file transfers between the first author and the transcribers were conducted through an encrypted file transfer system, and all transcribed texts were password protected. The transcribed text was double-checked against the audio recordings by the first author (WBJ) to ensure accuracy.

## Data analysis

We analysed the transcribed interviews using the qualitative content analysis method as described by **Graneheim & Lundman (2004)**. The interview texts were read in their entirety several times to establish a general understanding of the data. Three content areas were identified with little interpretation: a) description of ageing and healthy ageing; b) description of own alcohol use and its relationship with healthy ageing; c) description of various resources related to ageing and reducing alcohol consumption.

The main coder (WBJ) divided the text in each content area into meaning units according to their content and context, condensed and coded each meaning unit in relation to the study's aim. All codes were compared, and similar codes were grouped into distinct clusters which were then labelled to form subcategories. Related subcategories were then sorted and abstracted into categories. The analysis up until the formulation of manifest content (subcategories and categories) was conducted with the help of NVivo version 1.7 (**QSR International Pty Ltd, released September 2022**). Finally, the underlying meaning (latent content) of the categories was formulated into themes.

Even though the analysis process was inductive, it involved several comparisons between the whole and parts of the text to confirm the validity of codes, subcategories, categories, and themes. WBJ, FS, and ME held several meetings to reflect on the decontextualization (condensation and coding) and re-contextualization (sorting, abstraction, and interpretation) of the data, decide on category and theme formulation, and validate the preliminary results. In the final step, WBJ and AH met to review and validate the categories, subthemes, and themes, strengthening the credibility of the analysis.

## Ethical consideration:

Ethical approval for this study was obtained in advance from the Swedish Ethical Review Authority in Linköping (Dnr 2021-02240). Informed consent was obtained orally from the participants and recorded in accordance with the Helsinki Declaration. All data was collected and processed in accordance with the General Data Protection Regulation (GDPR) and after obtaining approval from Umeå University (PUR 2021/99). Participants were not provided any form of incentives.

## References

American Psychiatric Association. (2022). Diagnostic and statistical manual of mental disorders (5th , text rev. ed.).

Graneheim, U. H., & Lundman, B. (2004). Qualitative content analysis in nursing research: concepts, procedures and measures to achieve trustworthiness. *Nurse Educ Today*, *24*(2), 105-112. https://doi.org/10.1016/j.nedt.2003.10.001

Guest, G., Bunce, A., & Johnson, L. (2006). How Many Interviews Are Enough?: An Experiment with Data Saturation and Variability. *Field Methods*, *18*(1), 59-82. https://doi.org/10.1177/1525822X05279903

Malterud, K., Siersma, V. D., & Guassora, A. D. (2016). Sample Size in Qualitative Interview Studies: Guided by Information Power. *Qual Health Res*, *26*(13), 1753-1760. <https://doi.org/10.1177/1049732315617444>

QSR International Pty Ltd. (2022). *NVivo*. In (Version 1.7) [Computer software]. (QSR International Pty Ltd.
